# RETRACTION: Berberine Alleviates Contrast‐Induced Nephropathy by Activating Akt/Foxo3a/Nrf2 Signalling Pathway

**DOI:** 10.1111/jcmm.70863

**Published:** 2025-09-22

**RETRACTION:** W. Wang , R. Yu , C. Wu , Q. Li , J. Chen , Y. Xiao , H. Chen , J. Song , M. Ji , and Z. Zuo , “Berberine Alleviates Contrast‐Induced Nephropathy by Activating Akt/Foxo3a/Nrf2 Signalling Pathway,” Journal of Cellular and Molecular Medicine 28, no. 1 (2024): e18016, 10.1111/jcmm.18016.37909687 PMC10805492

The above article, published online on 01 November 2023 in Wiley Online Library (wileyonlinelibrary.com), has been retracted by agreement between the journal Editor‐in‐Chief, Stefan Constantinescu; The Foundation for Cellular and Molecular Medicine; and John Wiley and Sons Ltd. The retraction has been agreed upon following an investigation into concerns raised by a third party, regarding the accuracy of the flow cytometry analyses and apoptosis measurements in Figure 5A and 8B, as well as the integrity of the Western blot data in Figure 7 and 8C. The authors provided partial raw data, which revealed methodological issues, including inappropriate flow cytometer settings and incorrect use of the apoptosis detection kit, which renders the published flow cytometry results unsupported. Additionally, errors were identified in the representation of Western blot data in Figures 2B, 7, and 8. The authors fully cooperated with the investigation and repeated the relevant experiments. However, the new data failed to fully support the original conclusions, raised further concerns regarding the reproducibility of the flow cytometry results, and highlighted additional methodological issues in the Western blot analyses. Given the identified issues, the editors have lost confidence in the data presented and consider the conclusions of this manuscript insufficiently supported. The authors did not respond when asked to agree to the final wording of the retraction.

